# A parainfluenza virus 5 (PIV5)-vectored intranasal vaccine for Lyme disease provides long-lasting protection against tick transmitted *Borrelia burgdorferi* in mice

**DOI:** 10.21203/rs.3.rs-3143132/v1

**Published:** 2023-07-18

**Authors:** Maria Cristina Gingerich, Nisha Nair, J. Filipe Azevedo, Kamalika Samanta, Suman Kundu, Biao He, Maria Gomes-Solecki

**Affiliations:** 1 Department of Infectious Diseases, College of Veterinary Medicine, University of Georgia, Athens, GA, USA; 2 CyanVac, LLC, Athens, GA, USA; 3 Department of Microbiology, Immunology, and Biochemistry, University of Tennessee Health Science Center, Tennessee, USA; 4 Immuno Technologies, Inc., Memphis, TN, USA

## Abstract

Lyme disease (LD) is the most prevalent vector borne disease in North America and Europe and its geographic range continues to expand. Strategies for disease control are necessary to effectively reduce incidence of LD including development of safe vaccines for human use. Parainfluenza virus 5 (PIV5) vector has an excellent safety record in animals and PIV5-vectored COVID-19 and RSV vaccines are currently under clinical development. We constructed PIV5-vectored LD vaccine candidates expressing OspA from *B. burgdorferi* sensu stricto (OspA_B31_) and a chimeric protein containing sequences from *B. burgdorferi* and *B. afzelii* (OspA_BPBPk_). Immunogenicity and vaccine efficacy were analyzed in C3H-HeN mice after prime-boost intranasal (IN) vaccination with PIV5-OspA_B31_ and PIV5-OspA_BPBPk_, subcutaneous (SC) vaccination with rOspA_B31_+Alum as well as the respective controls. Mice vaccinated with either PIV5-A_B31_ or PIV5-A_BPBPk_ intranasally had high endpoint titers of serum antibody against OspA antigen beyond 1 year post vaccination, similar to levels detected in mice vaccinated SC with rOspA_B31_. Flowcytometric analysis of spleen cells at 9-months post-immunization demonstrated that immunization with the intranasal PIV5 vaccine candidates led to an overall increase in the number of memory B cells, cytotoxic T and cytotoxic effector T cells compared to SC groups. Borreliacidal activity measured by neutralization assay was maintained up to 18 months post-immunization, with the response greater in intranasal PIV5-delivered OspA vaccines, than that induced by SC rOspA_B31_. Challenge with infected ticks (10-19 strains of *B. burgdorferi*) performed at 4-, 9- or 15-months post-immunization showed increased breakthrough infections in mice vaccinated with SC rOspA_B31_ compared to IN PIV5-A_B31_ or IN PIV5-A_BPBPk_ at 9- and 15-months, as determined by qPCR of *B. burgdorferi* in tissues, culture of *B. burgdorferi* from tissues, and antibodies against *B. burgdorferi* protein VIsE. These data demonstrate that intranasal PIV5-based immunization is superior to parenteral immunization with the same recombinant protein and provides long-lasting protection (> 1 year) against Lyme disease.

## Introduction

Lyme disease (LD) is a tick-borne illness caused by the spirochete *Borrelia burgdorferi* sensu lato (Bbsl), (*Borreliella genus novum* under consideration ^1^). In humans, it is a progressive illness with a wide range of common clinical manifestations gradually developing from early to late stage. Late disseminated infection is associated with permanent damage to the nervous and musculoskeletal systems ^2 3 4 5^. LD is the most prevalent vector borne disease throughout the Northeast, Mid-Atlantic and Midwest regions of the U.S. where the disease is endemic and its range continues to expand^6^. Analysis of anonymous insurance claims data from 2010-2018 ^7^ led the CDC to estimate that ~ 476,000 people were diagnosed and treated for LD annually during that period. This new estimate further highlights the importance of LD as a public health concern. The most effective way to avoid this debilitating disease is to avoid tick-infested areas. Additional disease control strategies are necessary to effectively reduce incidence of Lyme disease including development of safe vaccines for human use.

Outer surface protein A (OspA) is the only immunogen proven to provide 76-92% protection ^8,9^ against tick-transmitted *B. burgdorferi* in fully vaccinated human subjects. After a 20-year hiatus, a re-engineered vaccine based on the C-terminus sequence of OspA from several Bbsl genospecies (VLA15) is undergoing Phase III clinical trials by Pfizer/Valneva ^10^ using a 3 shot scheme of intramuscular injections. Parainfluenza virus 5 (PIV5) is a non-segmented, negative-strand, RNA virus of the family *Paramyxoviridae* ^11^. PIV5 delivered vaccines have an excellent safety record ^12^ and have completed phase I clinical trials for COVID-19 and respiratory syncytial virus (RSV). Because PIV5 is a respiratory virus, it is a great vaccine vector for intranasal delivery, as it elicits protective mucosal IgA antibody responses locally, as well as systemic humoral and cell-mediated immune responses ^13^. We describe the construction of a PIV5-vectored LD vaccine expressing OspA from *B. burgdorferi* sensu lato and its immunogenicity and protective efficacy after challenge of intranasally vaccinated mice with *B. burgdorferi* sensu stricto infected ticks.

## Methods

### Construction of PIV5-vectored vaccines expressing OspA_B31_ and OspA_BPBPk_

#### Cells:

BHK21 cells were maintained in Dulbecco’s modified Eagle medium (DMEM) containing 10% tryptose phosphate broth (TPB), 5% fetal bovine serum (FBS), 100 IU/mL penicillin, and 100 μg/mL streptomycin (1% P/S; Mediatech Inc., Manassas, VA). Vero E6 and MBCK cells were maintained in Dulbecco’s modified Eagle media (DMEM) supplemented with 5% fetal bovine serum (FBS) plus 100 IU/mL penicillin and 100ug/mL streptomycin (1% P/S; Mediatech Inc, Manassas, VA, USA). All cells were incubated at 37°C, 5% CO_2_.

#### Viruses:

The PIV5-A_B31_ and PIV5-A_BPBPk_ plasmids, encoding the full-length genome of PIV5 and a *Borrelia burgdorferi* strain B31 OspA protein and well as BPBPk protein gene sequences ^21^ inserted between the PIV5 SH and HN genes was constructed as previously ^14^.Virus rescue was performed as described ^14^. Briefly, the PIV5-A_B31_ and PIV5-A_BPBPk_ plasmids and four helper plasmids—pPIV5-NP, pPIV5-P, pPIV5-L, and pT7-polymerase, encoding the NP, P, and L proteins and T7 RNA polymerase, respectively—were co-transfected into BHK21 cells at 90% confluence in 6-cm plates using JetPrime (Polyplus). Recovery of the virus is indicated by syncytia formation. The virus was then plaque-purified as a single plaque from BHK21 cells. The full-length genomes of the plaque-purified single clone of PIV5-A_B31_ and PIV5-A_BPBPk_ viruses were sequenced as described before ^14^. Viruses were grown in MDBK cells for 5 to 7 days using DMEM containing 2% fetal bovine serum (FBS). Media were collected and pelleted at 3000 rpm to remove cell debris by using a Sorvall tabletop centrifuge for 10 min. Virus supernatant was supplemented with 10% sucrose-phosphate-glutamate buffer, snap-frozen in liquid nitrogen, and stored at −80°C immediately after collection.

#### Western blot:

Immunoblotting was performed on Vero cells in 6-well plates that were infected with PIV5, PIV5-A_B31_, or PIV5-A_BPBPk_ at an MOI of 1. At 48 hours post-infection (hpi), Laemmli sample buffer (Bio-Rad, catalog no. 1610737) with 5% β-mercaptoethanol was used to lyse cells. The lysates were separated on an SDS–polyacrylamide gel electrophoresis (SDS-PAGE) gel and immunoblotted with a mouse anti–OspA antibody (184.1) and an anti–PIV5-NP antibody.

### Immunization, immunogenicity and vaccine efficacy analysis

**Figure 1.**
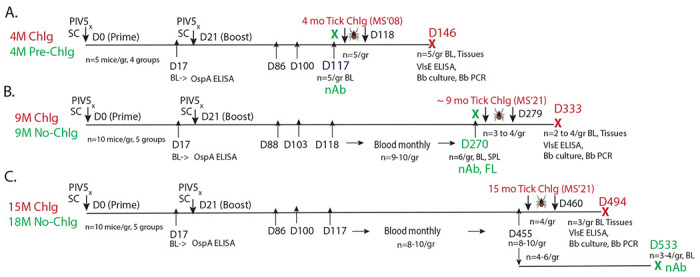
Vaccination schedule and methods for evaluation of efficacy before and after tick challenge at 4-months, 9-months and 15-months post prime. Legend: PIV5x, intranasal droplet immunization with PIV5 delivered vaccines; SC, subcutaneous inoculation; D, day after prime dose; Bb, *B. burgdorferi*; X, euthanasia; BL, blood; SPL, spleen; nAb, neutralization assay; FL, flow cytometry.

All experiments were performed in accordance with protocols approved by the IACUC at the University of Georgia (A2023 01-021-Y1-A0) and at the University of Tennessee Health Science Center (19-103). A graphic representation on the animal experimental design is shown in Figure 1.

#### Immunization:

Briefly, groups of 5 to 8-week-old female C3H-HeN mice (Envigo) were anesthetized and intranasally inoculated with 10^6^ PFU of PIV5 vector, PIV5-A_B31_, or PIV5-A_BPBPk_, or with 20 μg of rOspA_B31_+Alum subcutaneously. Twenty-one days after prime immunization, the mice were boosted with the same preparations. Blood (Fig. 1) was collected before tick challenge for determination of anti-OspA antibody on the following days: d17, d86, d100 and d117 for Study 1 (4-month challenge, D117 serum was used for neutralization assays); d17, d88, d103, d118 and monthly thereafter until d270 for Study 2 (9-month challenge, D270 serum was used for neutralization assays). For Study 3 (15-month challenge and 18-month neutralization), groups of mice were bled on d17, d86, d100 and d117 and monthly thereafter until D455 for 15-month challenge; a subset of these mice were not challenged and were kept until 18 months post prime-boost for an additional collection of blood at D533 for analysis of anti-*B. burgdorferi* neutralization activity.

#### Enzyme-linked immunosorbent assay (ELISA):

Anti-OspA and anti-*B. burgdorferi* VlsE antibody was determined by ELISA ^15^. Briefly, 5-10μg/ml of purified recombinant OspA or VlsE were coated on flat-bottom ELISA plates (Thermo Fisher Nunc MaxiSorp) and incubated at 4°C overnight. The following day, the plates were washed, blocked, and incubated with primary antibodies, i.e., serum (1:10^2^), followed by the secondary antibody HRP-conjugated goat anti-mouse IgG (Jackson ImmunoResearch, Inc) diluted at 1:10000. Endpoint titers of anti-OspA antibodies were calculated using serum (1:10^2^ to 1:10^6^) from study 3 (15-month challenge) collected at pre-boost, 3 months, 6 months and 12 months post-prime vaccination. Blood used for VlsE ELISA was collected at euthanasia on D146 (4 month challenge), D333 (9 month challenge) and D494 (15 month challenge).

#### Neutralization antibody (nAb) assay:

Briefly, blood was collected from groups of vaccinated mice the day before challenge (d117) for the 4-month challenge experiment, and on days 270 and 533 from groups of vaccinated mice that were not subjected to challenge for the 9 month and 18 month experiments, respectively. Neutralization of multi-strain cultures of *B. burgdorferi* by fresh serum was performed as described ^15^. Briefly, 8 μl of the bacterial culture was mixed with 4 μl of heat-inactivated mouse serum obtained from vaccinated and control mice and with 4 μl guinea pig complement (MP Biomedicals^™^) in a 0.2 ml sterile PCR tube (VWR, LLC Radnor, PA). The positive control group consisted of 8 μl BSK media (Sigma-Aldrich, Saint Louis, MO) with 8 μl *B. burgdorferi* culture. Samples were incubated at 34°C for 6 days. The cultures were counted in five fields on days 0, 2, 5, 7 (4-month) or 0, 3, and 6 (9- and 18-month) for viable, motile *B. burgdorferi* using a Petroff-Hausser chamber under a dark-filed microscope (Zeiss USA, Hawthorne, NY) and averaged to get the total number of live, motile *B. burgdorferi*. The final percentage survival was calculated in comparison to the Day 0 count for each sample.

#### Amplification of OspC by conventional PCR:

Five flat nymphal *Ixodes scapularis* ticks were crushed and DNA was extracted using the DNAeasy tissue kit as per the manufacturer’s recommendation (Qiagen, Valencia CA). OspC specific amplicons were prepared using primers (F)-AATAAAAAGGAGGCACAAATTAATG and (R)-GTAACTGGAAAAATAAAGTCAATAT by conventional PCR. Each 20 *μ*l PCR reaction mixture contained 200 *μ*M deoxynucleoside triphosphate (dNTP) (Thermo Scientific), 1 U Taq DNA polymerase (Thermo Scientific), 2 *μ*l of 10X Taq Buffer (Thermo Scientific), 2.5 mM MgCl_2_ and 0.4 *μ*M of each OspC primer, and 1 *μ*l of purified genomic DNA (gDNA). The reaction mixture was heated at 95°C for 4 min, amplified for 36 cycles at 95°C for 30 s, 58°C for 30 s, and 72°C for 60 s, and finally incubated at 72°C for 5 min. The PCR products (2-5 ul) were electrophoresed on a 2% agarose gel, pre-stained with 1:10,000 DNA SafeStain (Lambda Biotech-C138) and imaged under a UV gel doc system. AM-Pure beads were used to clean unused primers, dNTPs, and other reagents. The amplicon quantity was measured on a Qubit 4 fluorometer (Thermo Fisher Scientific, Waltham, MA, USA) using the Qubit dsDNA HS assay kit to ensure at least 10ng/*μ*l for library preparation as per the manufacturer’s recommendation. Amplicon quality was measured using a nanodrop instrument to confirm gDNA purity with A260/230 and A260/280 absorbance rations of at least 1.8 for all samples. Library preparation was done using NEBNext^®^ Fast DNA Fragmentation & Library Prep Set for Ion Torrent^™^. Samples were amplified and barcoded with barcode adapters from the Ion Xpress^™^ Barcode Adapters 1-16 Kit. Sequencing was done with the Proton Ion Torrent Sequencing instrument (ThermoFisher) at the UTHSC Molecular Resource Center.

#### OspC sequencing data analysis:

Fastq files were retrieved from the Ion Torrent Sequencing instrument. The individual runs were combined to create the merged fastq files. FastQC was used to check the quality of the reads. Reads were trimmed if needed using FASTX-Trimmer. Data was aligned against the fasta file containing OspC variants provided. SAM files were mined for total read count tables. SAM files were then filtered to obtain only uniquely aligned fragments. Filtered SAM files were mined for unique read count tables. Graphs were created using R ^16^.

#### Tick challenge:

Three colonies of infected *I. scapularis* were maintained in the laboratory. MS’08/NY derived from heart cultures from *Peromyscus leucopus* infected with field caught ticks (from New York State) between 2005 and 2008 and frozen at −80C; MS’21/MA derived from frozen cultures obtained from C3H-HeN mice inflected with ticks collected in MA parks in 2021; MS’21/NY derived from frozen cultures from C3H-HeN mice inflected with ticks collected in NY parks in 2021. Independent cultures were used to infect C3H-HeN mice; uninfected larval ticks were allowed to engorge in the infected mice and were allowed to molt to the nymphal stage before they were used for challenge. Challenge of experimental animals with *B. burgdorferi* was performed as described ^17^. Ticks from the MS’08/NY colony were used for the 4-month challenge experiment; equal numbers of ticks from each 2021 colony (MS’21/MA and MS/21/NY) were used for the 9-month and 15-month challenges. The *B. burgdorferi* infection prevalence of each tick colony was determined to be >80%. Briefly, 8-10 flat nymphal *Ixodes scapularis* ticks were placed between the ears of mice that were caged separately and held in FIC-2 isolators for a week. Engorged ticks that naturally fell off after taking a blood-meal were collected from the bottom of the cage, labeled, and stored at −20°C. Three weeks after the last day of challenge, mice were euthanized and blood, spleen, heart, joint and bladder were collected. Tissues were placed in BSK-H culture or RNAlater (Invitrogen, MA) for determination of *B. burgdorferi* viability (counting of live spirochetes under a dark field microscope) and dissemination (qPCR). Blood was used for analysis of anti-*B. burgdorferi* antibodies to rVlsE by ELISA and spleen was processed for isolation of splenocytes for flow cytometry analysis.

#### Quantitative PCR to enumerate *B. burgdorferi*.

Quantitative PCR (qPCR) was used to enumerate *B. burgdorferi* in BSK cultures from glycerol stock, heart cultures and *B. burgdorferi* load in tissues and ticks. Ticks and tissues (bladder, heart, joint) were processed for DNA extraction using the DNAeasy tissue kit as per the manufacturer’s recommendation (Qiagen, Valencia CA). The eluted DNA was stored at −20°C. qPCR was done on QuantStudio 3 (Applied Biosystems) using *B. burgdorferi* flaB primers, a known conserved gene of *B. burgdorferi* ^18^. The following are the sequences: forward GCAGCTAATGTTGCAAATCTTTTC, reverse GCAGGTGCTGGCTGTTGA, and probe [6~FAM]-AAACTGCTCAGGCTGCACCGG-[Tamra~Q]. For the standard curve, DNA from *B. burgdorferi* culture was purified and serially diluted from 10^5^ to 1 *B. burgdorferi*. The PCR reaction was performed using the fast advance master mix (Applied Biosystems^™^ Taqman^™^) in a final 20 μl volume which contained 25 μM of each primer, 250 nM of the specific probe, and 2 μl of DNA. PCR cutoff was set to C_t_ value >38.

#### Flow Cytometry.

Single cell suspensions from spleen were prepared on the day of euthanasia after RBC lysis using a previously described protocol with minor modifications ^19 20^. Dead cells were eliminated using Live/dead cell stain during cell counting using a Luna cell counter (Logos Biosystems, South Korea). Approximately 10^6^ cells were seeded per well in a 96 well microtiter plate and blocked with anti-mouse CD16/32 antibody (1:50) for 15-20 min on ice in staining buffer. Surface staining was performed using appropriate primary conjugated antibody against different cell surface markers and incubated in the dark for 30 min at 4°C. Cells were washed twice with phosphate buffered saline (PBS, 1X; pH 7.4) and fixed with 4% Paraformaldehyde for 10 mins followed by a single PBS wash. Cells were resuspended in staining buffer, acquired on BioRad ZE5 Cell analyzer and data were analyzed using Flow Jo software. The panel of fluorochrome conjugated antibodies is described in Supplementary Material (Table S1).

### Statistical analysis.

Data are represented as mean ± standard deviation. Statistical analysis was performed for each vaccine group by nonparametric test with Dunn’s multiple comparisons in comparison to pre-boost (Figs 4 and 5). Ordinary One-way ANOVA with uncorrected Fisher’s LSD was used to compare flow cytometric differences within groups (Fig 6). One-way ANOVA followed by Dunnett’s multiple comparison test by comparing groups to IN PIV5 group (Fig 8). GraphPad Prism was used for the statistical analysis and plotting the graphs.

## Results

### Generation and characterization of PIV5-based vaccines expressing the outer membrane protein A (OspA).

**Figure 2.**
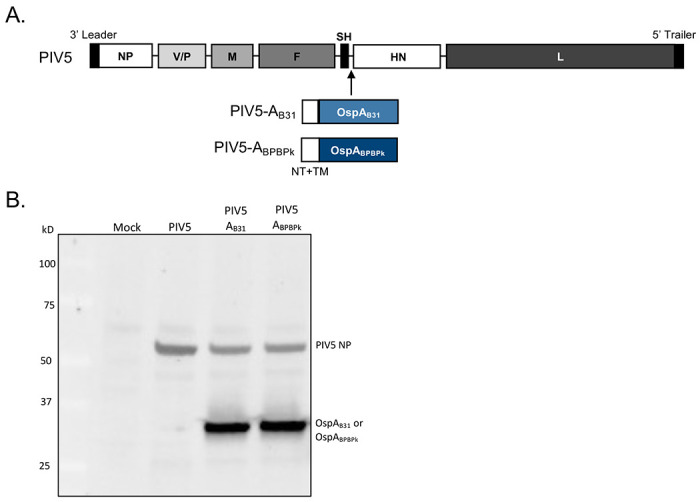
Generation and characterization of PIV5-A_B31_ and PIV5-A_BPBPk_ vaccine candidates. (A) Schematic of vaccine candidates. PIV5 has seven genes encoding for eight proteins, 3’ leader, NP, V/P, M, F, SH, HN, L, 5’ trailer. The OspA B31 and BPBPk proteins contain the N-terminus (NT) and transmembrane domain (TM) sequence from PIV5 HN protein. (B) Detection of OspA expression. Vero cells were mock-infected or infected with PIV5 vector control, PIV5-A_B31_, or PIV5-A_BPBPk_ at an MOI of 1. Forty-eight hours after infection the cells were lysed, and the lysates resolved on an SDS-PAGE gel and immunoblotted with anti-OspA and anti-PIV5 NP antibodies.

We generated the PIV5-A_B31_ and PIV5-A_BPBPk_ vaccine candidates by inserting the *ospA* gene sequence in the PIV5 genome between the SH and HN genes (Fig. 2A). PIV5-A_B31_ carried the full-length OspA sequence from *B. burgdorferi* strain B31. PIV5-A_BPBPk_ carried a full-length chimeric sequence of OspA in which the *B. burgdorferi* B31 amino acid sequences 165-189 and 219-273 were replaced with the respective sequences from *B. afzelii* strains (BPBPk) ^21^. The N-terminus and transmembrane domain sequences from the PIV5 HN protein were also introduced directly upstream of the *ospA* gene in both vaccine candidates to improve incorporation of the bacterial protein into the PIV5 virion. The vaccine viruses were rescued as previously described ^14^, and their genomes were confirmed through RT-PCR and sequencing. Expression of the OspA protein in PIV5-A_B31_- and PIV5-A_BPBPk_-infected cells was confirmed through western blot assay (Fig. 2B).

### Analysis of *B. burgdorferi* strain variability in *I. scapularis* used for tick challenge.

**Figure 3.**
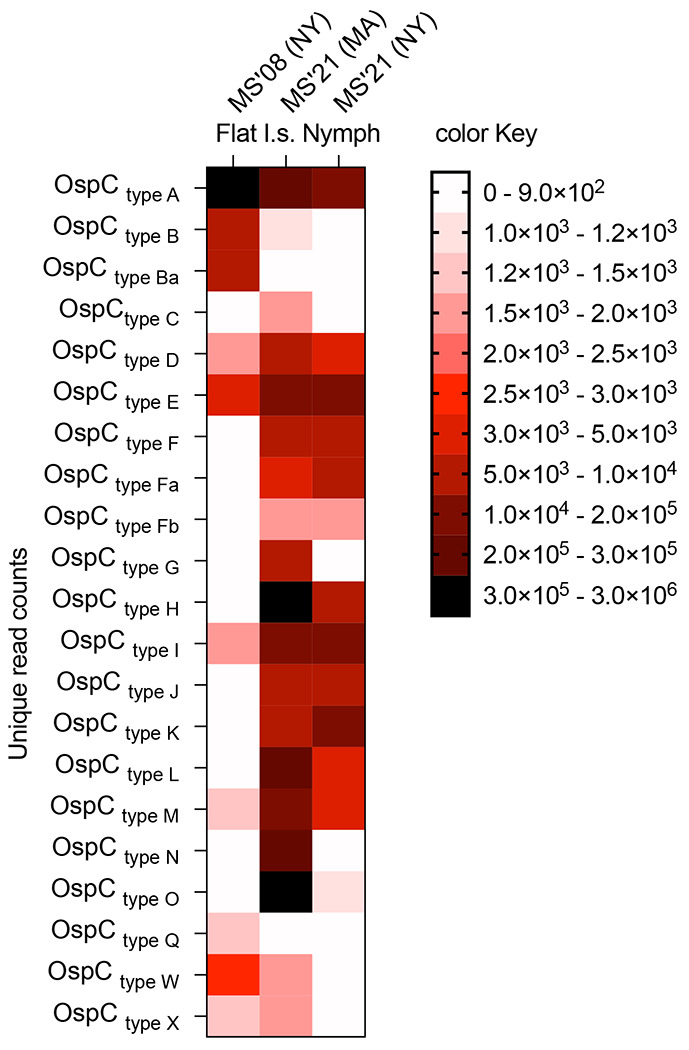
Heat map analysis of *B. burgdorferi* (*Bb*) strain variability in laboratory-maintained *I. scapularis* (I.s.) nymphal ticks by Proton Ion Torrent sequencing of the *ospC* gene. MS’08 (NY), MS’21 (MA) and MS’21 (NY)- the *Bb* culture used to produce the ticks originated from tissues from mice infected with ticks flagged in NY 2008, in MA 2021 and in NY 2021.

Sequencing OspC genes from the 3 colonies of *I. scapularis* ticks maintained in the laboratory for tick challenges (Fig. 3) showed that multi-strain MS’08/NY ticks mostly carried 10 types of OspC (A, B, Ba, D, E, I, M, Q, W, X) with >1000 unique read counts, whereas the other OspC types had <900 unique reads. MS’21/MA ticks mostly carried 19 types of OspC (A, B, C, D, E, F, Fa, Fb, G, H, I, J, K, L, M, N, O, W, X) with >1000 unique read counts, whereas the other two OspC types had <900 unique reads. MS’21/NY ticks mostly carried 13 types of OspC (A, D, E, F, Fa, Fb, H, I, J, K, L, M, O) with >1000 unique read counts whereas the other OspC types had <900 unique reads.

### Immunization with PIV5-A_BPBPk_ or PIV5-A_B31_ induces long-lasting neutralizing antibody responses.

**Figure 4.**
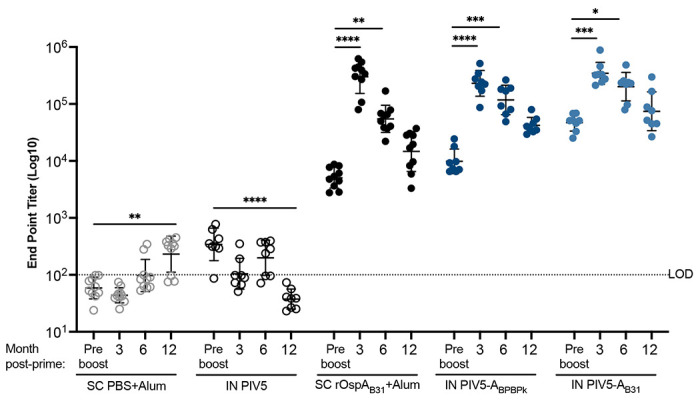
Intranasal vaccination with PIV5-A_BPBPk_ or PIV5-A_B31_ induces long-lasting humoral immune response. Mice in study 3 (15-month challenge) received two doses of alum alone or alum + 20 μg of rOspA_B31_ protein subcutaneously, or 10^6^ PFU of PIV5 vector control, PIV5-A_BPBPk_, or PIV5-A_B31_ intranasally at 21 days interval. Blood collected at day 17 and months 3, 6, or 12 post-prime were used. Anti-OspA_B31_ IgG antibody titers were quantified by ELISA. The LOD is indicated by the dotted line. Statistical significance was calculated for each vaccine group by nonparametric test with Dunn’s multiple comparisons in comparison to pre-boost. (*p≤0.05, **p< 0.01, ***p< 0.001, ****p< 0.0001).

To assess humoral IgG immune responses induced by the PIV5-A_BPBPk_, PIV5-A_B31_ and SC OspA_B31_ vaccines, all blood collected before tick challenge was used for determination of anti-OspA antibody (1:100) by ELISA. In contrast to the controls, serum from mice vaccinated with OspA (IN and SC) collected before (D17) and after the boost (>D86) generally had anti-OspA IgG antibody OD_450_ > 3.5. To quantify anti-OspA IgG endpoint titers we used serum from Study 3 (15-month challenge) collected at day 17 (pre-boost), and at months 3, 6 and 12 post-prime (Fig 4) diluted at 10^2^-10^6^ on ELISA. Mice from the SC rOspA_B31_+Alum, PIV5-A_BPBPk_, and PIV5-A_B31_ vaccine groups had peak serum IgG end point titers (EPT) at 3 months post-prime, with geometric means of 5.5, 5.5 and 5.4 log_10_, respectively. These values were significantly higher than serum IgG EPTs from day 17 post-prime, showcasing the boosting effect by the second dose of the vaccine. These levels of anti-OspA_B31_ serum IgG antibodies decreased over the next 9 months of the experiment, with mice from the SC rOspA_B31_+Alum group having the biggest decrease of 1.7 log_10_ EPT compared to 0.7 log_10_ EPT seen in mice from the PIV5-A_BPBPk_ and PIV5-A_B31_ vaccine groups. Mice from the SC PBS+Alum and IN PIV5 control groups had very low levels of cross-reactive IgG antibodies at some point throughout the study, but the peak titer for these control groups are 3 log_10_ lower than the peak titers for all vaccine groups. These results suggest that immunization with an intranasal, PIV5-based vaccine may lead to longer-lasting protection than a protein-based vaccine given subcutaneously.

**Figure 5.**
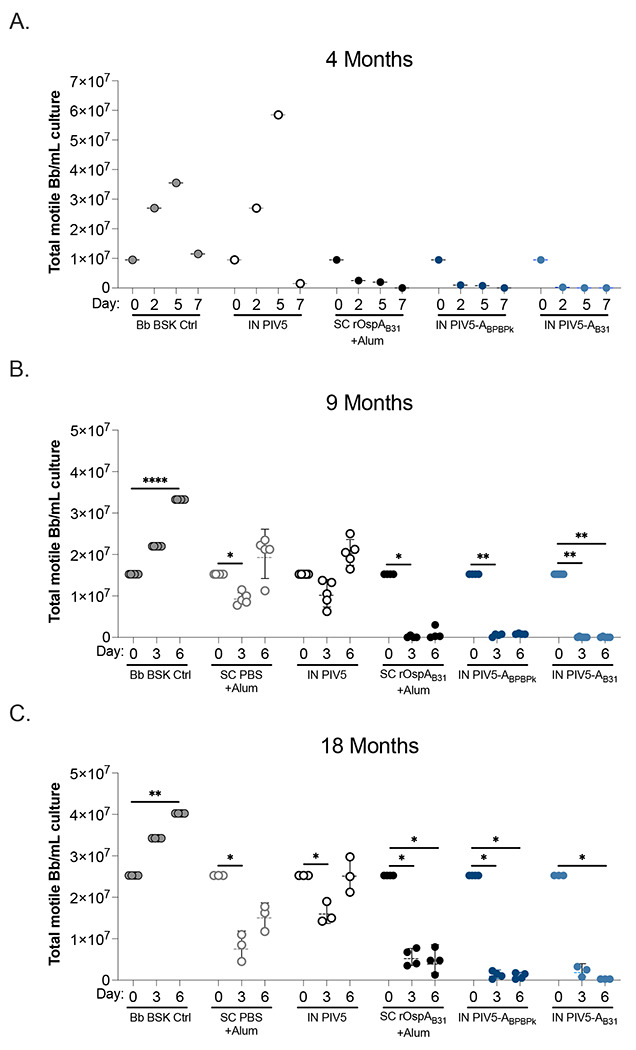
Neutralization of *B. burgdorferi* in culture. Serum from vaccinated mice was collected before tick challenge at 4 months after prime vaccination on D117 (A), at 9 months after prime vaccination on D270 (B) and at 18 months after prime vaccination on D533 (C). Serum from the 4-month experiment was pooled per group (n=5 mice/group) because the volume per individual was insufficient. For subsequent experiments (9-month and 18-month), serum from n=3-6 mice per group were individually tested. Statistical significance was calculated for each vaccine group by nonparametric test with Dunn’s multiple comparisons in comparison to day 0. (*p≤0.05, **p<0.01, ****p< 0.0001). Bb, *Borrelia burgdorferi*.

Similar to study 3, mice from study 2 (9-month, no-challenge) were immunized with either two-doses of alum alone (SC PBS+Alum) or alum plus 20 μg of rOspA protein (SC rOspA_B31_+Alum) subcutaneously, or with two-doses of 10^6^ PFU of wild-type PIV5 (IN PIV5), PIV5-A_BPBPk_, or PIV5-A_B31_ intranasally (Fig. 1). Study 1 contained the same groups, except for the SC PBS+Alum group. For this study blood was collected on D117 for neutralization assays of *B. burgdorferi* in culture (Fig. 5) before the mice were challenged (4-month, pre-challenge). Because insufficient blood was collected from each mouse, we pooled the serum from each group for the neutralization assay (Fig. 5A). For the longevity studies (Studies 2 and 3) we vaccinated additional groups of mice to do neutralization assays, thus sufficient serum was collected at euthanasia from each mouse on D270 (Study 2, 9-month, no challenge, Fig. 5B) and D533 (Study 3, 18-month, no-challenge, Fig. 5C). Total motile bacteria were measured at days 0, 2, 5, and 7 post neutralization for study 1, and days 0, 3, and 6 for studies 2 and 3. At 4-months after prime-boost vaccination (Study 1), the levels of motile bacteria in cultures incubated with serum from the SC rOspA_B31_+Alum, PIV5-A_BPBPk_, and PIV5-A_B31_ vaccine groups decreased by 1.6, 0.6, and 1.0 log_10_, respectively at day 2 compared to day 0 (Fig. 5A). These values further decreased on day 5 until they reach 0 for all vaccine groups at day 7 post-neutralization (Fig. 5A). In contrast, the Bb BSK and IN PIV5 control groups had increasing number of motile bacteria at days 2 and 5 post-neutralization, with peak numbers at day 5 post-inoculation with geometric means of 7.6 and 7.8 log_10_ of motile *B. burgdorferi* per milliliter of culture, respectively. These values decreased slightly on day 7 post-neutralization to 7.1 and 6.2 log_10_ of motile *B. burgdorferi* per milliliter of culture, respectively. At 9-months after prime-boost immunization (Study 2), vaccine groups SC rOspA_B31_+Alum, PIV5-A_BPBPk_, and PIV5-A_B31_ show a significance decrease in numbers of motile bacteria in cultures at day 3 or 6 post-inoculation compared to day 0, with almost all samples from each group reaching 0 at day 3 post-neutralization (Fig. 5B). On the other hand, samples from the Bb BSK, SC PBS+Alum, and IN PIV5 control groups had an increase in the levels of motile bacteria in cultures on day 6 post-neutralization compared to day 0. On average, levels of motile *B. burgdorferi* per milliliter of culture in control groups increased by 0.2 log_10_ on day 6 post-neutralization compared to day 0. At 18-months post prime-boost vaccination (Study 3), the data continues to show a significant reduction in levels of motile *B. burgdorferi* per milliliter of culture in samples from the SC rOspA_B31_+Alum, PIV5-A_BPBPk_, and PIV5-A_B31_ vaccine groups at days 3 and 6 post-neutralization compared to day 0, with the largest difference seen on day 6 with an average decrease of 0.8, 1.5 and 2.0 log_10_, respectively (Fig. 5C). In contrast, the Bb BSK and IN PIV5 control groups had either an increase or no change in numbers of motile *B. burgdorferi* per milliliter of culture at day 6 post-neutralization compared to day 0, with a 0.2 log_10_ growth seen in the Bb BSK samples. These results demonstrate that an intranasal PIV5-based vaccine carrying OspA sequences from different *B. burgdorferi* sensu lato genospecies can generate robust neutralizing IgG immune responses that can last for up to 18 months, and these responses are higher than those seen with a recombinant protein-based vaccine.

### Immunization with PIV5, PIV5-A_BPBPk_ and PIV5-A_B31_ induces robust cellular immune responses.

**Figure 6.**
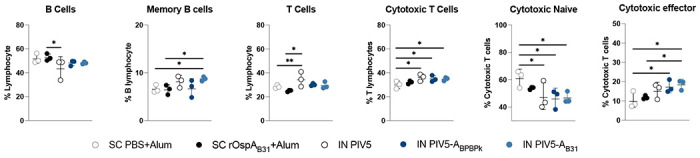
Flowcytometric analysis of immune cells from mice vaccinated intranasally with PIV5 delivered OspA vaccines compared to SC rOspA delivered in alum. Spleen samples were analyzed by flow cytometry from 9-month post-vaccinated mice that did not undergo tick challenge. Representative graphs show differences in frequency of B and T cell types between control and vaccinated groups. Statistical analysis by Ordinary One-Way ANOVA with uncorrected Fisher’s LSD. (*p≤0.05, **p<0.01).

Cell-mediated immune responses induced by PIV5, PIV5-A_BPBPk_ PIV5-A_B31_ and SC rOspA_B31_+alum vaccines were assessed trough flowcytometric analysis of splenocytes. Mice from the 9-month study (study 2) were humanely euthanized on D270 and spleen was collected for flow cytometry. Figure 6 shows the percentage of B cells, memory B cells, T cells, cytotoxic T cells, cytotoxic naïve T cells, and cytotoxic effector T cells induced by each vaccine candidate. Data shows that immunization with the PIV5 vaccine candidates (including PIV5 control) lead to an overall increase in the number of memory B cells, cytotoxic T and cytotoxic effector T cells compared to SC PBS+Alum groups (Fig. 6). Of note, significantly higher percentage of memory B cells were quantified in spleen from the PIV5-A_B31_ vaccinated group (8.7%) compared to the SC rOspA_B31_+Alum group (6.6%). For cytotoxic T cells, both the PIV5-A_BPBPk_ and PIV5-A_B31_ vaccine groups had a significantly higher percentage of cells, with 34.9% and 34.8%, respectively compared to 29.9% in the SC PBS+Alum control group. Lastly, the levels of cytotoxic effector T cells in both PIV5-A_BPBPk_ and PIV5-A_B31_ vaccine groups were significantly higher than the SC PBS+Alum group, with 17.1%, 18.4%, and 9.7% of cytotoxic effector T cells in each group respectively. Furthermore, animals from the PIV5-A_B31_ vaccine group had a significantly higher percentage of cytotoxic effector T cells (18.4%) compared to the SC rOspA_B31_+Alum group, which only had 11.8%. Together, these results demonstrate that the PIV5-based vaccine candidates can induce long-lasting cell-mediated immune responses, that at 9 months post prime-boost, are higher than SC alum adjuvanted vaccines and that these responses are associated with intranasal administration of the virus.

### PIV5-A_BPBPk_ and PIV5-A_B31_ provide long-term protection against tick challenge with multiple strains of *B. burgdorferi* up to 15 months post prime-boost immunization.

**Figure 7.**
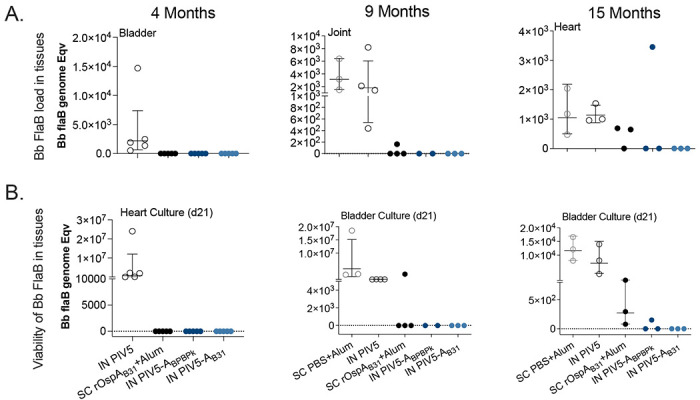
Analysis of *B. burgdorferi* dissemination to tissues after tick challenge of mice vaccinated intranasally with PIV5 delivered OspA vaccines compared to SC OspA delivered in alum. Bladder, heart and joint tissues were collected from all vaccinated mice euthanized at 4 months, 9 months and 15 months post prime vaccination and processed for Bb DNA purification and *flaB* qPCR. Bladder and heart tissues were also cultured in BSK-H media for analysis of *B. burgdorferi* viability and samples were collected for confirmation by PCR. Representative graphs are shown for Bb load in tissues (A) and viability of Bb in tissues (B). Bb, *Borrelia burgdorferi.*

Mice from the 3 studies were challenged with ticks infected with multiple strains of *B. burgdorferi* at 4-months (Study 1), 9-months (Study 2), or 15-months (Study 3) post-prime. Figure 2 shows a heat map analysis of the *B. burgdorferi* OspC type variability used in each study, MS’08 (NY) for Study 1 and MS’21 (MA/NY) for Studies 2 and 3. To access *B. burgdorferi* dissemination to target tissues, bladder, joint, and heart samples were tested for *B. burgdorferi* FlaB load by qPCR (Fig. 7A). Furthermore, heart and bladder tissues were cultured in BSK-H medium to evaluate mobility/viability of *B. burgdorferi* under a dark field microscope (Table 1) that was confirmed by FlaB PCR from the cultures (Fig. 7B). In Study 1, the challenge done 4 months after prime-boost vaccination with SC rOspA_B31_, IN PIV5-A_BPBPk_ and IN PIV5-A_B31_ resulted in absence of *B. burgdorferi* FlaB DNA in heart, bladder and joint, as well as absence of viable *B. burgdorferi* in cultures from heart, in contrast to the controls that received intranasal (IN) PIV5 or subcutaneous (SC) PBS+alum. In Study 2, the challenge done 9 months after prime-boost vaccination with IN PIV5-A_BPBPk_ and IN PIV5-A_B31_ resulted in absence of *B. burgdorferi* FlaB DNA in heart, bladder and joint, as well as absence of viable *B. burgdorferi* in cultures from bladder, in contrast to 1/4 mice that received SC rOspA_B31_ and all controls that received intranasal (IN) PIV5 or subcutaneous (SC) PBS+alum. In Study 3, the challenge done 15 months after prime-boost vaccination with IN PIV5-A_B31_ resulted in absence of *B. burgdorferi* FlaB DNA in heart, bladder and joint, as well as absence of viable *B. burgdorferi* in cultures from bladder, in contrast to 1/3 IN PIV5-A_BPBPk_, 3/3 mice that received SC rOspA_B31_, and all controls that received intranasal (IN) PIV5 or subcutaneous (SC) PBS+alum.

**Figure 8.**
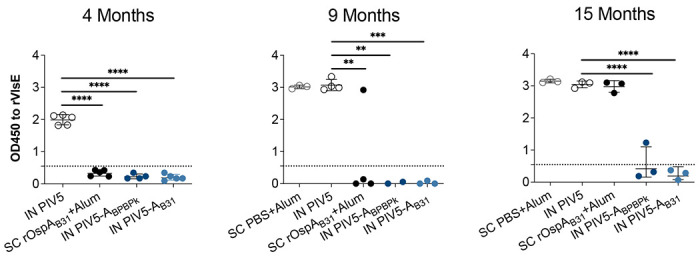
Serologic evidence of *B. burgdorferi* dissemination after tick challenge of mice 4 months, 9 months and 15 months post vaccine prime. Serum IgG to recombinant *B. burgdorferi* VlsE was determined by ELISA. Error bars: mean with SD; Statistical significance was calculated through one-way ANOVA followed by Dunnett’s multiple comparison test by comparing groups to IN PIV5 group. (**p<0.01, ***p<0.001, ****p<0.0001).

To further confirm *B. burgdorferi* dissemination after challenge, IgG to *B. burgdorferi* VlsE protein was quantified in serum from vaccinated mice, by ELISA (Fig. 8). In Study 1 (4-month challenge), mice vaccinated with IN PIV5-A_BPBPk_, IN PIV5-A_B31_, and SC rOspA_B31_+Alum had anti-VslE IgG antibody OD_450_ values below the limit of detection, in contrast to the IN PIV5 control group. In Study 2 (9-month challenge), mice vaccinated with IN PIV5-A_BPBPk_ and IN PIV5-A_B31_ vaccine groups also had anti-VlsE IgG antibody OD_450_ values below the limit of detection, while 1/4 mice from the SC rOspA_B31_+Alum group had detectable levels of anti-VslE IgG antibodies. Lastly, in Study 3 (15-month challenge), mice from the IN PIV5-A_B31_ vaccine group continue to have anti-VslE IgG antibody OD_450_ values below the level of detection, while 1/3 mice from the IN PIV5-A_BPBPk_ and 3/3 mice from the SC rOspA_B31_+Alum groups had detectable levels of anti-VslE IgG antibodies in the serum. Together, these data demonstrate that an intranasal PIV5-based Lyme disease vaccine can provide considerably longer-lasting protection than a protein-based vaccine given subcutaneously in the mouse model. The entire set of data is summarized in Table 1.

## Discussion

Currently, the most effective way to prevent Lyme disease is to avoid *I. scapularis* tick infested areas. This is unfeasible for those who work outside and for those who enjoy spending quality time outdoors in the Spring and Summer in endemic areas. Thus, there is a pressing need for development and commercialization of effective and acceptable vaccines to control Lyme disease. We developed a novel parainfluenza 5 (PIV5) viral-vectored vaccine for intranasal delivery that provides long-lasting protection against tick-transmitted *B. burgdorferi* using a prime-boost scheme of immunization.

Outer surface protein A from *B. burgdorferi* sensu stricto (OspA) is the only immunogen proven to provide 76% (LYMErix^™^) ^8^ and 92% (ImuLyme^™^) ^9^ protection against tick-transmitted *B. burgdorferi* in fully vaccinated human subjects after 3 intramuscular injections. One of the two vaccines (LYMErix^™^) was approved by the FDA in 1998. Although analysis of adverse effects performed in both clinical trials showed no significant increase in the frequency of arthritis events between vaccine and control groups ^8 9^, some individuals who received LYMErix, reported developing arthritis after the trial period ended ^22^. Furthermore, within weeks of the clinical trial reports, another study suggested that a cross-reactive autoimmune event between an epitope in *B. burgdorferi* OspA and a human integrin (hLFA-1) might drive the inflammatory response in the joints of some treatment-resistant Lyme arthritis patients ^23^. Even though LYMErix was never linked to causing arthritis, demand for this vaccine decreased substantially and the product was taken off the market in 2002 ^24^. Keeping all these factors in mind, we designed a chimeric sequence of OspA by replacing the hLFA-1 partially homologous epitope within *B. burgdorferi* OspA with the analogous sequence from *B. afzelii* (a non-arthritogenic *Borrelia* species) to generate the new OspA_BPBPk_ construct. This sequence was cloned into a Lactobacillus expression vector and was shown to prevent tick-transmitted *B. burgdorferi* infection in mice, thus providing an effective oral vaccine candidate for Lyme disease ^21^.

Parainfluenza virus 5 (PIV5) is a non-segmented, negative-strand, RNA virus and a member of the *Rubulavirus* genus of the family *Paramyxoviridae*. PIV5 is a promising safe viral vaccine vector. Live PIV5 has been part of the kennel cough vaccine for dogs for 50 years, yet no disease has ever been reported due to human exposure to vaccinated dogs ^25^. Because PIV5 does not have a DNA phase in its life cycle, its use avoids the unintended consequences of genetic modifications of host cell DNA through recombination or insertion. Various recombinant PIV5 viruses expressing GFP or immunogens have been generated and shown to be genetically stable ^26^. In addition, PIV5 can grow in Vero cells at titers greater than 10^8^ PFU/mL, making vaccine production cost effective. PIV5-based influenza (IAV)^14,27–31^, respiratory syncytial virus (RSV)^13,32,33^, MERS-CoV^34^ and, most recently SARS-CoV-2^35^, vaccines are efficacious in various preclinical animal models. RSV and COVID-19 vaccines have recently completed phase 1 clinical trials.

In this study, we demonstrate that both PIV5-based vaccines, PIV5-A_BPBPk_ and PIV5-A_B31_ administered in a homologous prime-boost vaccination regimen intranasally, can induce robust humoral IgG immune responses in mice reaching over 5 log10 EPT for serum IgG antibodies at 3 months post-vaccination, with these values moderately decreasing up to 12 months post-immunization (Fig. 4). The longevity of the immune response can also be seen in Fig. 5, where neutralization of *B. burgdorferi* with mouse serum was shown to be significant up to 18 months post-immunization for both PIV5-A_BPBPk_ and PIV5-A_B31_ vaccine groups. The serum IgG antibody response seen in PIV5-A_B31_-vaccinated mice seems to be slightly higher than the one seen in PIV5-A_BPBPk_-vacinated mice; this could be due to the use of a recombinant rOspA_B31_ protein instead of a rOspA_BPBPk_ protein to coat the ELISA plates. One of the advantages of using live replicating viral vectored vaccines is that IgG responses are more durable ^36^ than responses induced by mRNA vaccines which usually wane within 6 months to 1 year ^37^. For OspA recombinant proteins, others have shown that OspA-specific IgG antibody also starts to decline 3-6 months after the 2^nd^ or 3^rd^ dose within the first year of vaccination ^38^. Other schedules of administration (3 shots within 2 months) produced anti-OspA antibody responses that could only be protective for one tick-season (about 4 months) ^39^. Furthermore, Comstedt et al, reported that immunization with 3 doses of VLA15 in mice resulted in a robust serum IgG antibody response, but the levels of antibodies decreased more than 10-fold, five months after immunization ^10^. A booster dose was administered 5 months after immunization to increase these levels. To accommodate these issues, the VLA15 vaccine undergoing phase 3 clinical trials requires a 3-shot intramuscular vaccination schedule on Day 1, Day 57 and Day 180. Here we show that a mucosal delivered viral-vectored vehicle leverages a non-invasive administration route (intranasal) and generates a rapid, durable and neutralizing IgG response to the immunogen that lasts up to 18 months. We also found that this IgG antibody response was accompanied by increased memory B cells and cytotoxic effector T cell populations that appear to be driven by PIV5 (Fig. 6). PIV5-based vaccines have been shown to induce robust levels of cellular immunity that aid in protection against bacterial and viral pathogens in various animal models^13,32,40^, further validating the use of a live, viral-vectored vaccine platform for vaccine development. Furthermore, longevity of protection was associated with PIV5 vaccinations in challenges performed at 9 and 15 months post-prime vaccination (Fig. 7, Fig. 8 and Table 1), with PIV5 delivered OspA_B31_ providing 100% (0/6) protection and PIV5 delivered OspA_BPBPK_ providing 67% (2/6) protection, whereas SC immunizations with recombinant OspA_BPBPk_ resulted in 0% (6/6) protection, after challenges were done with ticks infected with 13-19 distinct *B. burgdorferi* types (by OspC sequencing, Fig. 3). Differences in vaccine efficacy between the PIV5 delivered OspAs may be attributed to homologous vaccination versus challenge (OspA_B31_ from *B. burgdorferi* B31 v *B. burgdorferi* sensu stricto challenge) and heterologous vaccination versus challenge (OspA_BPBPk_ containing sequences from *B. burgdorferi* and *B. afzelii* v *B. burgdorferi* sensu stricto challenge).

### Conclusion:

We developed a novel PIV5-vectored OspA-based intranasal vaccine that prevents tick-transmitted *B. burgdorferi* infection after challenge at 4-, 9- and 15-months post prime-boost immunization. Our work advances the field of development of vaccines for Lyme disease in three ways: i) it reduces the number of immunizations, ii) it simplifies administration from the classic intramuscular injection to a novel intranasal mist with possibility for self-administration, iii) and most importantly, it extends protection from ~ 4 months to 15 months post-immunization.

## Figures and Tables

**Table 1.** Data summary.

Study	Groups	Route of inoculation	No. of mice	Bb baseline nAb No. pos/No. tested live Bd in culture	baseline Immune cells in pLN (Hi/Lo)	Bb flab load in tissue after challenge No. pos/total (%)	Bd viability after challenge (culture motility DFM)	Bb VlsE serology after challenge
**4m**	PIV5	10^6^ PFU IN	5	5/5 - 5/5	NA	5/5	5/5	5/5
	rOspA_B31_+Alum	20 μg SC	5	0/5 - 0/5	NA	0/5	0/5	0/5
	PIV5-A_BPBPk_	10^6^ PFU IN	5	5/5 - 0/5	NA	0/5	0/5	0/5
	PIV5-A_B31_	10^6^ PFU IN	5	5/5 - 0/5	NA	0/5	0/5	0/5
**9m**	PBS+Alum	SC	10	5/5 - 5/5	Ctrl	3/3	3/3	3/3
	PIV5	10^6^ PFU IN	10	5/5 - 5/5	Hi T Cytotoxic	4/4	4/4	3/3
	rOspA_B31_+Alum	20 μg SC	10	1/4 - 3/4	No difference	1/4	1/4	3/3
	PIV5-A_BPBPk_	I10^6^ PFU IN	10	3/4 - 4/4	Hi T Cytotoxic Hi T Cytotoxic Effector	0/2	0/2	1/3
	PIV5-A_B31_	10^6^ PFU IN	10	1/6 - 1/5	Hi Memory B Cells Hi T Cytotoxic Hi T Cytotoxic Effector	0/3	0/3	0/3
**15m**	PBS+Alum	SC	10	3/3 - 3/3	NA	3/3	3/3	3/3
	PIV5	10^6^ PFU IN	10	3/3 - 3/3	NA	3/3	3/3	3/3
	rOspA_B31_+Alum	20 μg SC	10	4/4 - 4/4	NA	2/3	3/3	3/3
	PIV5-A_BPBPk_	10^6^ PFU IN	10	4/4 - 4/4	NA	1/3	1/3	1/3
	PIV5-A_B31_	10^6^ PFU IN	10	3/3 - 3/3	NA	0/3	0/3	0/3

***Bb, B. burgdorferi***

## Data Availability

All data generated or analyzed during this study are included in this manuscript.
